# Glucosamine inhibits extracellular matrix accumulation in experimental diabetic nephropathy

**DOI:** 10.3389/fnut.2022.1048305

**Published:** 2022-12-01

**Authors:** Loic Teuma, Rachana Eshwaran, Ulrich Tawokam Fongang, Johanna Wieland, Feng Shao, Maria Luisa Lagana, Yixin Wang, Ane Agaci, Hans-Peter Hammes, Yuxi Feng

**Affiliations:** ^1^Experimental Pharmacology Mannheim, Medical Faculty Mannheim, European Center for Angioscience (ECAS), Heidelberg University, Mannheim, Germany; ^2^Preclinical and Translational Pharmacology, Department of Pharmacy, Health and Nutritional Sciences, University of Calabria, Rende, Italy; ^3^5th Medical Clinic, Medical Faculty Mannheim, Heidelberg University, Mannheim, Germany

**Keywords:** glucosamine, diabetes, kidney, alpha-smooth muscle actin, glomerular expansion, endothelial cells, extracellular matrix

## Abstract

**Introduction:**

Glucosamine, the intermediate metabolite of the hexosamine biosynthesis pathway (HBP), is widely used as a supplementary drug in patients with osteoarthritis. However, its consequences in such patients concomitantly suffering from diabetic nephropathy is unknown.

**Methods:**

The aim of the study was to investigate the effect of exogenous administration of glucosamine in the diabetic kidney. A mouse model of streptozotocin-induced diabetic nephropathy *in vivo* and cultured endothelial cells *in vitro* were used in the study. The mice were treated with glucosamine for 6 months. Renal function was evaluated by metabolic cage, and histology of the kidney was estimated by periodic acid-schiff (PAS) staining. The expression of related genes was assessed by real-time PCR, immunofluorescence staining, immunoblotting and ELISA.

**Results:**

There was no significant difference in urinary albumin secretion, relative kidney weight, or creatinine clearance between the groups treated with glucosamine and controls. Assessment of the kidney demonstrated reduction in mesangial expansion and fibronectin expression in the diabetic glomeruli treated with glucosamine. Glucosamine treatment significantly decreased α-smooth muscle actin (α-SMA) protein expression in both diabetic and control kidneys, whereas the expression of other fibrosis-related genes and inflammatory factors was unaltered. Moreover, α-SMA colocalized with the endothelial marker CD31 in the diabetic and control kidneys, and glucosamine reduced α-SMA+ ECs in the diabetic glomeruli. In addition, glucosamine suppressed α-SMA expression in endothelial cells treated with or without high glucose.

**Discussion:**

In summary, this is the first report to show that glucosamine reduces mesangial expansion and inhibits endothelial-mesenchymal transition in diabetic nephropathy. The underlying mechanisms need to be further investigated.

## Introduction

Diabetic nephropathy (DN) is a common chronic complication of diabetes mellitus, affecting around 30 and 40% of patients with type 1 and 2 diabetes mellitus, respectively (1). DN is characterized clinically by increased albuminuria and decreased glomerular filtration, and morphologically by podocyte loss, thickening of the glomerular basement membrane (GBM) and expansion of mesangium and advanced glomerulosclerosis (2). Renal fibrosis is not only the pathognomonic feature, but also the common end route of DN. Its feature includes deposition of extracellular matrix in glomeruli, in the GBM, and in the tubulointerstitium (3, 4). The fibrotic changes are marked by increased expression of growth factors such as transforming growth factor β (TGF-β) and connective tissue growth factor (CTGF) as well as matrix proteins like collagen, α-SMA, and fibronectin. Several studies have shown that the development of fibrosis in the diabetic kidney is mainly dependant on the TGF-β1 signaling pathway that regulates the deposition of collagen, fibronectin, and laminin in the extracellular matrix in the GBM, and the mesangium (5, 6). TGF-β1 is also able to induce α-smooth muscle actin (α SMA) and fibronectin, and stimulate glomerular hypertrophy (7, 8). Additionally, it was found that renal endothelial cells are able to develop into matrix-producing myofibroblasts. The correlation between activation of myofibroblasts and deposition of fibrotic proteins has been proven (9). In particular, high expression of α-SMA was noted during the endothelial mesenchymal transition (EndoMT) in DN. The pathological alterations were hypothesized to be the result of activated intracellular signaling pathways involved in DN, such as the formation of advanced glycation end products (AGEs), the hexosamine biosynthesis pathway (HBP), the polyol pathway, as well as the activation of protein kinase C (PKC) (10–12).

Glucosamine is a physiologically occurring sugar molecule in the HBP and an essential component of glycoproteins, proteoglycans, and glucosaminoglycans (13). It is formed biologically in the HBP from fructose-6-phosphate, an intermediate product of glycolysis. Through the HBP, glucosamine is converted to UDP-N-acetylglucosamine (UDP-GlcNAc), which can enter GlcNAc cycle of proteins, resulting in regulation of protein functions (14). UDP-GlcNAc is also a precursor for the production of glucosaminoglycan chains bound to proteoglycans which are essential components of the GBM, extracellular matrix and endothelial glycocalyx, involving in many physiological and pathological processes in the kidney (15). Glucosamine is also a food supplement frequently utilized therapeutically in the treatment of osteoarthritis and arthritic complaints (16). Glucosamine has been shown to possess anti-inflammatory, anti-fibrotic, and anti-oxidative features (17–19).

Nevertheless, the exogenous supply of glucosamine has a controversial effect on the kidney. It has been reported that exogenous glucosamine supply can increase flux through the HBP, resulting in a consequential increase in posttranslational O-GlcNAc modifications of proteins, lipids, and nucleic acids in the cell, and this increase was identified as an important mediator for the development of diabetic complications, particularly DN (10). Kolm-Litty et al. showed that glucosamine strongly increased the expression and activation of TGF-β in cultured mesangial cells. The effect of glucosamine on TGF-β expression was more potent than that of glucose, resulting in increased production of matrix components leading to fibrosis (5). In addition to increasing TGF-β expression, other studies demonstrated an elevation of the plasminogen activator inhibitor 1 (PAI-1) expression in mesangial cells by glucosamine, providing a possible explanation for the glomerular hypertrophy via activation of the HBP (20, 21). In contrast, Park et al. showed an anti-fibrotic, anti-oxidative, and antiproliferative effect of glucosamine in the kidneys, observing a reduction in the expression of α-SMA, fibronectin, and collagen after treatment with glucosamine in a model of non-diabetic renal fibrosis. A down-regulation of the TGF-β signaling pathway *in vitro* was also demonstrated in that study (18). Hu et al. showed that despite activation of the HBP leading to enhanced protein O-GlcNAcylation, glucosamine supplementation resulted in a renoprotective effect via inhibition of oxidative stress and apoptosis (22). The finding suggest that the kidney may be affected by exogenous glucosamine independent of O-GlcNAc.

Exogenous glucosamine is able to be administered intravenously, intraperitoneally or orally. Our published data on oral glucosamine in diabetic retinopathy showed that glucosamine had no effect on water and food intake, urinary excretion, feces production, also blood glucose and HbA1c in diabetic animals compared with controls. However, glucosamine supplementation resulted in a significant body weight gain in normal control but not in diabetic mice. Unexpectedly, exogenous glucosamine promoted vascular damage in the non-diabetic retina, but, at the same time, it protected neuronal function in the diabetic retina (23).

Based on the role of glucosamine, basic knowledge of the HBP and pathomechanisms of DN, we studied the effect of exogenous glucosamine supplementation on DN using the same diabetic animals induced by STZ.

## Materials and methods

### Animals

All animal experiments in this study were approved by the local ethics committee (Regierungspräsidium Karlsruhe, G178/15) Karlsruhe Regierungspräsidium and carried out in C57Bl/6 male mice. The animals had free access to food and water and were cared by trained staff. The animals were randomly divided into 4 groups, NC: normal control group: NC+G: normal mice treated with glucosamine; DC: diabetic control group; DC+G: diabetic mice treated with glucosamine. Diabetes was induced by intraperitoneal injection of streptozotocin (STZ, 145 mg/kg body weight) into approximately 10-week-old mice. A successful induction of diabetes was defined when blood glucose was more than 250 mg/dl 1 week after STZ injection. The glucosamine was mixed into the standard food (SSNIFF, Soest, Germany) which was supplied to the control mice. The mixture of the glucosamine was performed in the company SSNIFF. Oral glucosamine treatment via food [10 g/kg body weight, (19, 23)] was commenced 1 week after successful induction of diabetes, and continued for 24 weeks. Mice were injected subcutaneously with insulin (Lantus) three times a week if they lost more than 10% of body weight after being diabetes, and the general condition was not good. At the end of the experiments, the mice were sacrificed by isoflurane followed by cervical dislocation, and the kidneys were removed for further experiments.

### Metabolic cage and analysis of urine and serum

At the end of the experiments, the mice were weighed in the late afternoon and placed in metabolic cages for 16 h to assess the general metabolic parameters and renal function. The mice had free access to food and water in the cages. In the metabolic cages, food and water intake, urinary excretion, and feces weight were determined. Additionally, serum was collected and stored at –80°C for further analysis. Measurement of urinary albumin, creatinine, and urea was performed on a Hitachi 917 Autoanalyser (Roche Diagnostics GmbH, Mannheim, Germany). Simultaneously, levels of blood glucose, HbA1c, creatinine and urea were measured in the plasma.

### Periodic acid-schiff staining of the kidney

Periodic acid-schiff (PAS) staining was used to evaluate the histology and mesangial expansion in the renal cortex. Paraffin sections of kidney (4 μm) were dewaxed at 60°C for 1 h and deparaffinized in Roti-Histol and hydrated in a descending ethanol series. Then, the sections were place in 1% periodic acid for 10 min and Schiff’s reagent for 10 min followed by a staining with Mayer’s Hematoxylin for 1 min. After washing in an increasing ethanol series and incubation in Roti-Histol, the sections were embedded with Entellan. Samples were observed with a Leica microscope and images were taken with 40x objective, and analyzed using ImageJ. The mesangial expansion area (FMA = fractional mesangial area) was calculated as the proportion of the area of the mesangial matrix to the total area of the glomerulus and presented in percentage as described by Rangan and Tesch (24).

### Immunofluorescence staining

Paraffin sections were used for the immunofluorescence assessment of α-SMA and CD31. Following a similar deparaffinization and hydration procedure as previously described, the antigens were unmasked using a citrate retrieval buffer. Cryosections were used for the staining of fibronectin and fixed with acetone. Unspecific binding was blocked with a 1% BSA blocking buffer and the cell membranes were permeabilized with 0.5% Triton-X 100 for 1 h at room temperature. The sections were further incubated with the primary antibody mouse anti-α-SMA (1:500 Sigma A5228) and endothelial marker CD31 (1:200 Santa Cruz sc-1506-R) for colocalization, or rabbit anti-mouse fibronectin (1:200 Sigma F3648) overnight at 4°C. After washing on the second day, samples were incubated with the corresponding goat anti-mouse secondary antibody labeled with Alexa Fluor 488 (1:200, Thermofisher A-11001) and/or swine anti-rabbit TRITC (1:20, Dako R0156) for 1.5 h, then counterstained with DAPI. Finally, the sections were covered with Roti FluorCare mounting medium and images were taken using a confocal microscope (Leica, Germany). The quantification of the immunofluorescence staining in the samples was done according to the protocols described in Rangan and Tesch (24). Briefly, the images from 20 glomeruli from each mouse were quantified using ImageJ software. A threshold was set on the grayscale images such that only the areas of immunofluorescence in the glomeruli were highlighted. A freeform selection was then made around the glomerulus, and the intensity of the immunofluorescence staining, the area of the glomerulus, the area of the immunofluorescence staining, and the co-localization of α-SMA and CD31 in glomerulus was measured and further evaluated. All the staining for quantification of fibronectin and collagen 4 were repeated in three individual mice in each group.

### Cell culture

Human umbilical vein endothelial cells (HUVECs) were isolated from fresh umbilical cords with agreement of patients according to a previously described protocol. The use of HUVECs was approved by the local medical ethics committee (Medical Faculty Mannheim, University of Heidelberg, Germany, 2012-338N-MA). HUVECs were cultured in ECBM containing 10% FCS in a 1% gelatin-coated cell culture dish. HUVECs until passage 4 were used for the experiments. 24 h after seeding cells for the experiments, HUVECs were serum-starved in ECGM with 0.5% FCS for 24 h followed by stimulation with high glucose (30 mM D-glucose) with or without glucosamine in 0.5% FCS for additional 24 h.

### Protein isolation and immunoblotting

Proteins from the renal cortex or HUVECs were extracted in RIPA buffer with proteinase inhibitors, and protein concentrations were determined using the BCA assay. Twenty-five microgram of proteins were separated by SDS-PAGE and transferred onto PVDF membranes. After blocking with Roti-block (Roth, Karlsruhe, Germany) for 1 h at room temperature, the membranes were incubated with primary antibodies overnight at 4°C and corresponding secondary antibodies for 1 h at room temperature. The proteins were visualized using a chemiluminescent peroxidase substrate (Roche, Mannheim, Germany; or Thermo Scientific, Rockford, USA). Protein expression was quantified using Image J (NIH, USA). Specific primary antibodies used: mouse-anti-α-SMA (Sigma-Aldrich, A5228, 1:2,000), goat-anti-CTGF (Santa Cruz, sc-14939, 1: 200), mouse-anti-γ-tubulin (Sigma, T6557, 1:10,000). The secondary antibodies conjugated with horseradish peroxidase: rabbit-anti-goat (Sigma-Aldrich, A8919), horse-anti-mouse (Cell Signaling, 7076, 1:20,000).

### Enzyme-linked immunosorbent assay

The proteins dissolved in RIPA buffer were used for detecting IL-1β levels in the kidney cortex. For this experiment, the IL-1β ELISA kit for sandwich ELISA from R&D Systems (MLB00C) was employed according to the manufacturer’s instructions. In brief, proteins were pipetted into a 96-well plate coated with the IL-1β antibody. After incubation for 2 h at room temperature, the plate was washed to remove unbound proteins. The secondary antibody coupled with enzyme was pipetted into the wells and incubated for 2 h at room temperature. Finally, the substrate was added into the wells and the reaction was stopped by a stop solution. The plate was read with a photometer and the data was evaluated.

### Real-time quantitative polymerase chain reaction

Real-Time Quantitative PCR were carried out as described previously (25). In brief, RNA was isolated from kidney cortex using TRIzol (Invitrogen, Karlsruhe, Germany) and the concentration of total RNA was measured. Then, RNA was transcribed into cDNA using the SuperScript™ VILO™ cDNA synthesis kit (Thermo Fischer Scientific, Germany) according to the manufacturer’s instructions. Further, Taqman analysis was performed using the Taqman 2x PCR Master mix (Applied Biosystems, Weiterstadt, Germany) in a QuantStudio3 real-time PCR system (Applied Biosystems by Thermo Fisher Scientific). The gene expression was calculated using the ΔΔ-CT method using β-Actin as housekeeping control (26). All primers and probes labeled with MGB-FAM for amplification were purchased from Thermo Fisher Scientific, β-Actin: Mm00607939_s1; α-SMA: Mm01546133_m1; Collagen 4a3: Mm00483669_m1; Fibronectin: Mm01256744_m1; IL-1β: Mm00434228_m1; TNF-α Mm00443259_g1.

### Statistical analysis

The data are presented as mean ± SEM. The results were compared using an analysis of variance. *Post hoc* intergroup was compared with the Tukey correction. GraphPad Prism 5 (GraphPad Software, La Jolla, CA, USA) was utilized for the statistical analysis. *p* values < 0.05 were considered significant.

## Results

### Glucosamine diminishes mesangial expansion in diabetic mice

**FIGURE 1:**
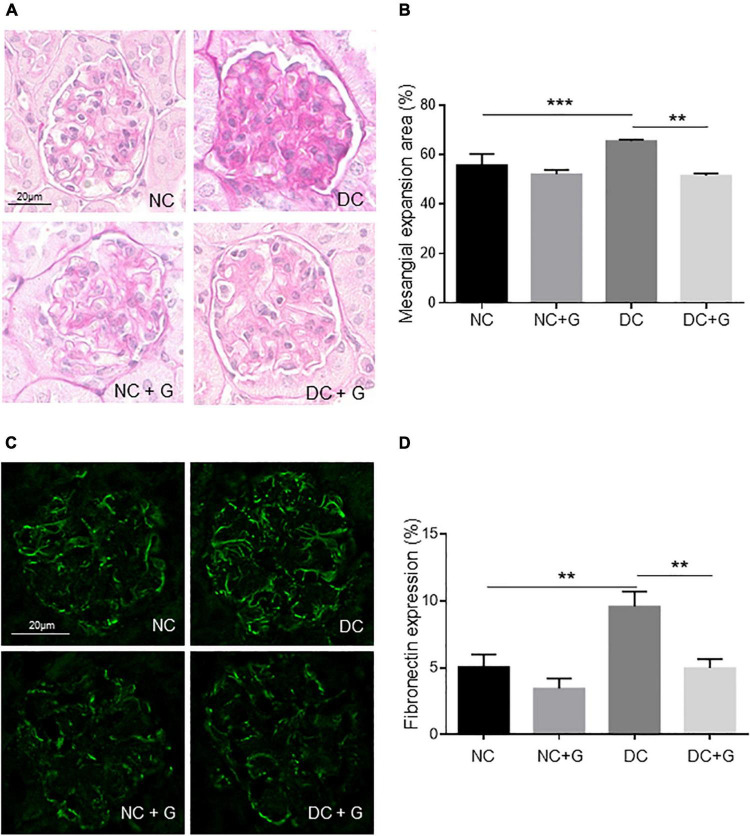
Glucosamine decreases mesangial expansion in diabetic kidneys. **(A)** Representative renal histology in diabetic mice treated with glucosamine. Kidney sections were stained with periodic acid-schiff (PAS). The PAS positive stained mesangial matrix was assessed and presented in panel **(B)**. **(C)** Immunofluorescence staining of fibronectin in the glomeruli of non-diabetic and diabetic mice treated with and without glucosamine. **(B)** Quantification of the fibronectin expression. Data are presented in mean ± SEM, n is 5 for **(B)**, and 20 for **(D)** fibronectin expression was verified in three individual serials. **p < 0.01, ***p < 0.001.

Diabetic nephropathy is characterized by progressive accumulation of extracellular matrix in glomerular. Thus, in this study, PAS-stained kidney sections were used to investigate the mesangial expansion in the glomeruli. As shown in Figures 1A,B, glomerular PAS staining is generally weak in the mesangial regions in the NC control groups, but enhanced considerably in the DC groups. The quantification results showed that the mesangial expansion area was significantly larger in the DC group compared to the NC group (*p* < 0.001). Treatment with glucosamine led to a significant (21%) reduction in the formation of the mesangial matrix in diabetic mice (*p* < 0.01). In the control mice, there was no significant change between animals with and without glucosamine treatment. Furthermore, we estimated the expression of fibronectin and collagen 4 using immunofluorescence staining. Fibronectin expression was significantly promoted by hyperglycemia. Glucosamine significantly reduced the expression of fibronectin elevated by hyperglycemia (Figures 1C,D). However, hyperglycemia-induced collagen 4 expression was not affected by the glucosamine supplementation (data not shown).

Since elevated glucosamine resorption into plasma upon glucosamine supplementation in diabetic and control mice was demonstrated in our previous publication (23), the data indicate that supplementation of glucosamine in diabetic nephropathy might abate the accumulation of mesangial extracellular matrix in diabetic conditions.

### Glucosamine does not alter the kidney function in diabetic mice

**FIGURE 2:**
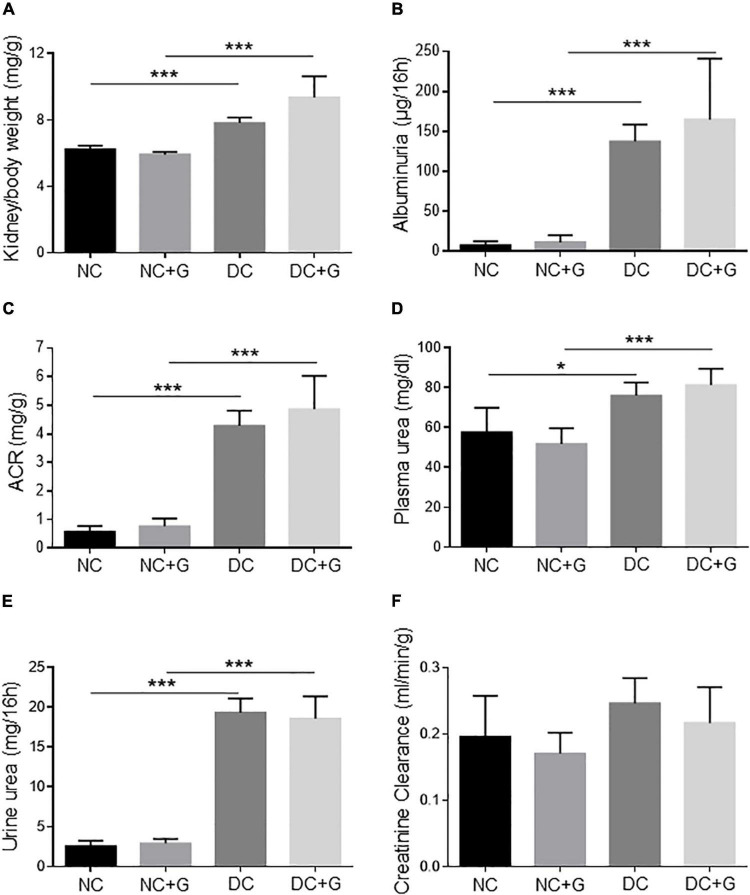
Glucosamine does not alter renal function in diabetic mice. **(A)** Kidney/body weight, **(B)** albuminuria, **(C)** albumin creatinine ratio, **(D)** plasma urea, **(E)** urine urea, and **(F)** creatinine clearance are determined. Mean ± SEM, *n* = 6–10, *p < 0.05, ***p < 0.001.

Firstly, we investigated the effect of glucosamine on kidney function by assessment of kidney weight and ratio of kidney weight to body weight between the groups (Figure 2A). The kidney weights did not differ between the four groups, with or without glucosamine treatment. The ratio of kidney to body weight was significantly higher in the diabetic DC group than in the control NC group (*p* < 0.001), suggesting hypertrophic alteration in the diabetic kidney. Nevertheless, treatment with glucosamine did not significantly change the ratio in either NC or DC animals.

Increased renal protein excretion, particularly albumin excretion, is a typical pathological feature of DN. In order to investigate its excretion, the albumin level was determined in the urine collected during the 16h metabolic cage (Figure 2B). Albumin excretion of DC animals was significantly higher than the NC group (*p* < 0.001). Supplementation of glucosamine did not change albumin excretion in either diabetic or non-diabetic mice. No changes in ACR upon glucosamine treatment were detected (Figure 2C).

Blood urea concentration (Figure 2D) and urine urea (Figure 2E) were significantly elevated in the diabetic animals whereas the creatinine concentration in the plasma of DC animals was comparable to that of the NC animals. After application with glucosamine, no significant difference was detected either in the normal or in the diabetic mice. In our DN model, creatinine clearance (Figure 2F) was slightly higher in the DC group than that in the NC group, however, it did not reach statistical significance. The filtration rate in the normal and diabetic kidneys remained unchanged by glucosamine.

### Glucosamine does not influence the transcriptional gene expression of extracellular matrix components and inflammatory factors

**FIGURE 3:**
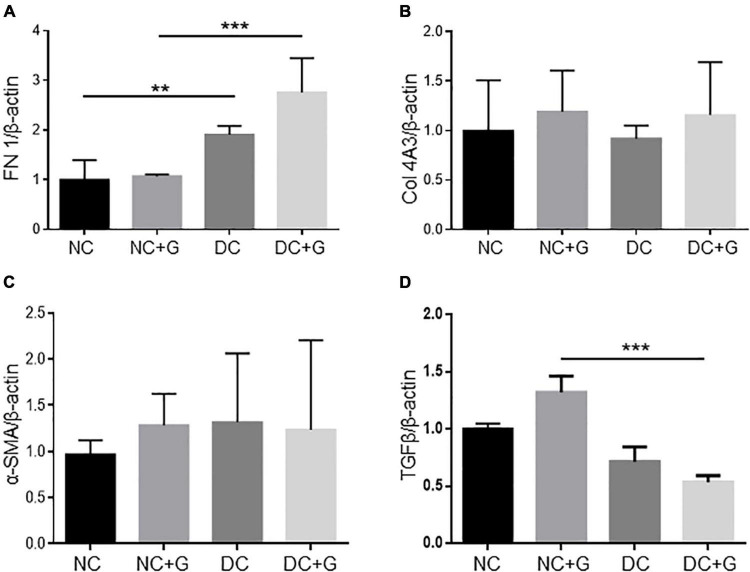
Fibrotic gene expression in diabetic kidneys is unchanged upon glucosamine treatment. Real-time PCR analysis for fibrotic genes **(A)** fibronectin (FN-1), **(B)** collagen 4A3 (Col 4A3), **(C)** α-SMA, and **(D)** TGFβ is shown. β-actin served as loading control. Each column shows mean ± SEM. *n* = 6. **p < 0.01, ***p < 0.001.

Studies reported that the elevated gene expression of extracellular matrix components contributes to the progression of diabetic nephropathy. Thus, we further addressed the transcriptional gene expression of FN-1, Collagen 1A1, 3A1, 4A1, 4A3, α-SMA, and TGF-β in the renal cortex using quantitative real time PCR (Figure 3). FN-1 expression was significantly increased in the DC group in comparison to the NC group (*p* < 0.01). Nevertheless, glucosamine showed no effect on the RNA expression of FN-1 in the kidney (Figure 3A). Unexpectedly, the mRNA expression levels of Collagen 1A1, 3A1, 4A1, and 4A3 were regulated neither by hyperglycemia nor by glucosamine (Figure 3B, data for Collagen 1A1, 3A1, 4A1 not shown). Moreover, there was no differences in the expression of α-SMA between the DC and NC groups, and the supplementation of glucosamine did not alter its expression in either non-diabetic or diabetic conditions (Figure 3C). Since TGF-β has been accepted to be the key growth factor mediating mesangial expansion, we further evaluated its expression level in the glucosamine treated renal cortex. In diabetic kidneys compared with non-diabetic controls, we observed a trend of downregulation in TGF-β by hyperglycemia in non-treated mice, but a significant decrease in glucosamine-treated mice which is likely due to a slight increase in TGF-β levels in glucosamine-treated controls. However, glucosamine did not change TGF-β expression levels either in basal or in diabetic conditions (Figure 3D).

Another aspect of diabetic nephropathy is the immigration of inflammatory cells and the release of inflammatory proteins. According to the feature of glucosamine in inhibiting inflammatory factors, the effect of glucosamine on inflammatory factors such as IL-1ß and TNF-α was also investigated by quantitative real time PCR (data not shown). The mRNA expression of TNF-α in diabetic kidneys tended to be elevated, however, did not reach significance compared to the control kidneys. Its expression was comparable in the groups with or without glucosamine treatment. Unexpectedly, IL-1β mRNA expression in the diabetic cortex tended to be lower than that in the NC group (*p* = 0.06). Glucosamine treatment showed no effect on its expression levels in the diabetic as well as in the non-diabetic kidneys. To verify the observation in IL-1β mRNA expression, we also assessed the IL-1β expression by ELISA using the cortex lysates, detecting a similar expression pattern with even a trend toward protein downregulation with hyperglycemia and glucosamine treatment.

### Glucosamine lowers the protein expression of α-smooth muscle actin

**FIGURE 4:**
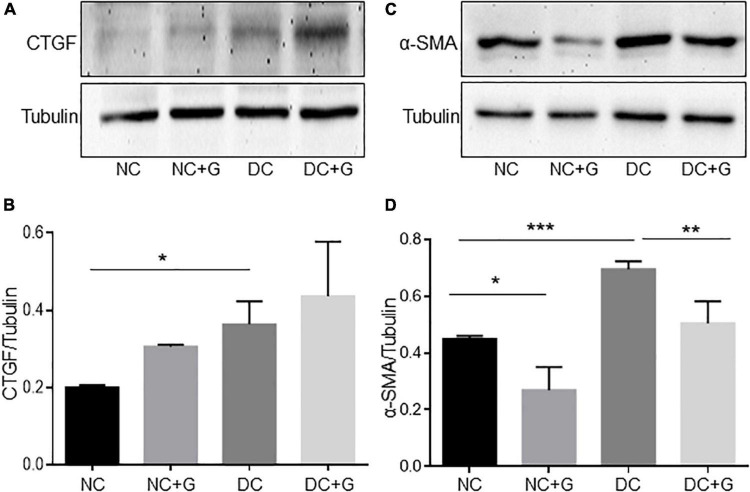
Glucosamine lowers protein expression of α-SMA in diabetic kidneys. Expression of CTGF and α-SMA in the renal cortex was assessed by Western blot **(A,C)** and quantified using ImageJ (**B**,**D)**. γ-tubulin served as loading control. Data are shown in mean ± SEM. *n* = 4. *p < 0.05, **p < 0.01, ***p < 0.001.

Based on the fact that glucosamine reduced the accumulation of extracellular matrix and expression of fibronectin in the glomeruli, but the transcriptional gene expression of extracellular components and inflammatory factors were not changed upon glucosamine treatment, we examined the protein expression of different markers for renal fibrosis such as fibronectin, collagen, connective tissue growth factor (CTGF), and α-smooth muscle actin (α-SMA) using immunoblotting (Figure 4). The western blot experiments using renal cortex lysates showed that there were no alterations in the expression of fibronectin and collagen either with hyperglycemia or glucosamine (data not shown). The CTGF protein expression was significantly higher in the DC group than in the NC group (p < 0.05), However, we could not detect any effect of glucosamine treatment on CTGF expression in either basal or diabetic conditions. We also analyzed whether the accumulation of mesangial matrix is associated with the expression alteration in α-SMA. The expression of α-SMA in the diabetic kidneys was significantly (54%) higher than in the control kidneys (*p* < 0.001), while it was moderately expressed in the control kidney. Glucosamine therapy significantly (27%) decreased α-SMA expression in both diabetic (*p* < 0.01) as well as in non-diabetic mice (*p* < 0.05).

**FIGURE 5:**
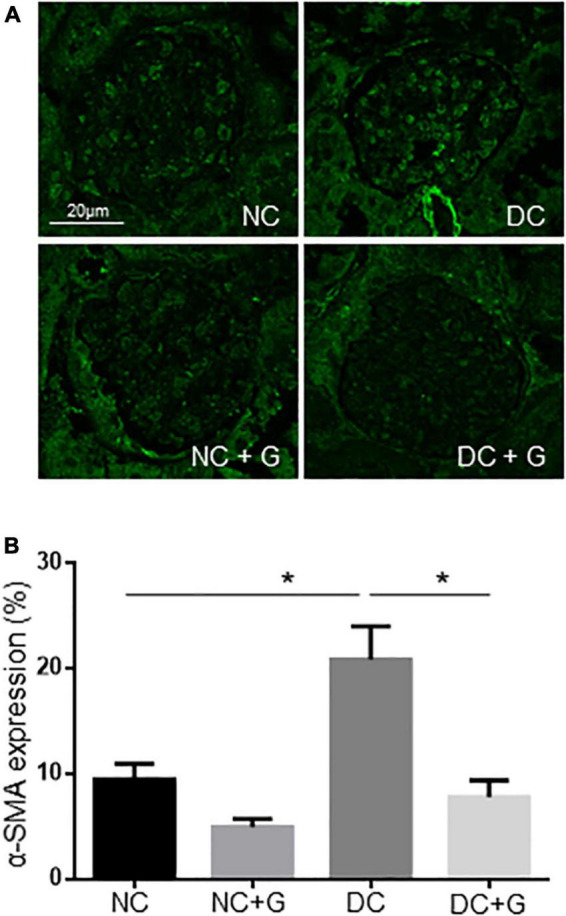
Glucosamine decreases α-SMA expression in the diabetic glomeruli. **(A)** Representative images show α-SMA expression detected by immunofluorescence staining. **(B)** Quantification of α-SMA expression was normalized by the glomerular areas from analyzing 20 glomeruli each kidney. The experiments were repeated in 3 kidneys each group. Data are shown in mean ± SEM. *n* = 20. *p < 0.05.

To confirm this result from the western blot and to know whether α-SMA is regulated in glomeruli as well, immunofluorescence staining of the kidneys with α-SMA antibodies was performed (Figure 5). A significant increase in the expression of α-SMA was observed in the DC group compared with the NC control group. In the diabetic groups treated with glucosamine, the expression of α-SMA was significantly weaker than in the DC group. The results suggest an increased α-SMA expression in the glomeruli of diabetic mice. Importantly, glucosamine lowered the expression of α-SMA in diabetic conditions. Additionally, combining the expression data of transcriptional and translation regulation of α-SMA, it suggests a regulation of α-SMA in translational levels rather than in transcriptional levels.

### Glucosamine decreases the number of α-SMA+ endothelial cells in diabetic nephropathy

**FIGURE 6:**
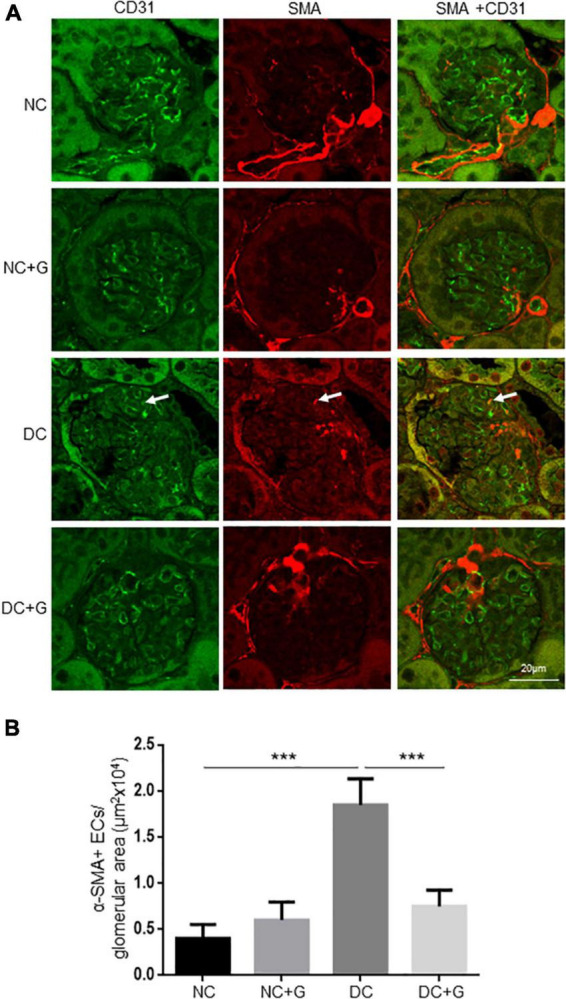
Glucosamine decreases the number of α-SMA+ endothelial cells in diabetic nephropathy. **(A)** Representative images are shown from double immunofluorescence staining with α-SMA and marker for endothelial cells CD31. **(B)** Quantification of α-SMA+ endothelial cells normalized to the glomerular areas (20 glomeruli analyzed per kidney). The experiments were repeated in 3 kidneys each group. Arrows indicate the illustrative co-localization of α-SMA and CD31. Data are shown in mean ± SEM. *n* = 20. ***p < 0.001.

Upon discovering the regulation of α-SMA expression by glucosamine in the glomeruli, next, we assessed the cell-specificity. We performed experiments colocalizing α-SMA with podocyte marker Nephrin. However, no obvious colocalization of α-SMA and nephrin was identified (data not shown). We also performed immunofluorescence staining using α-SMA and a cell marker of endothelial cells (CD31) (Figure 6). Unexpectedly, co-localization of α-SMA and CD31 was occasionally detected in the glomeruli in non-diabetic kidneys, suggesting that some endothelial cells exhibit certain features of mesenchymal cells in the normal kidney. Glucosamine did not change the numbers of α-SMA+ endothelial cells in the controls. Hyperglycemia facilitated their co-localization in the glomeruli compared with non-diabetic controls significantly. Glucosamine significantly (59%) diminished the hyperglycemia-prompted counts of α-SMA+ endothelial cells in the diabetic glomeruli.

**FIGURE 7:**
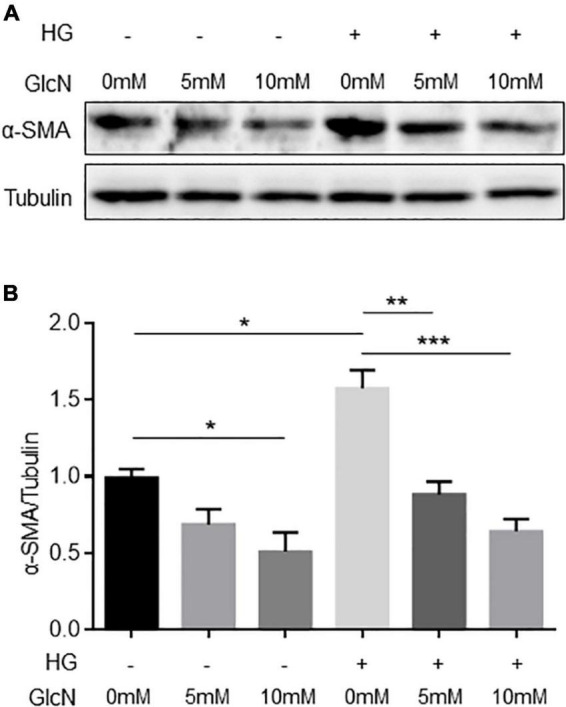
Expression of α-SMA in endothelial cells treated with high glucose and glucosamine. **(A)** A representative immunoblot is shown. **(B)** Expression of α-SMA was quantified using ImageJ. γ-tubulin served as loading control. Data are shown in mean ± SEM. *n* = 3. *p < 0.05, **p < 0.01, ***p < 0.001.

Furthermore, to confirm the *in vivo* findings, we evaluated whether glucosamine modulates the expression of α-SMA in endothelial cells *in vitro*. HUVECs were stimulated with and without high glucose and treated with glucosamine. In accordance with the observation showing α-SMA+ endothelial cells in the control glomeruli *in vivo*, the immunoblotting results demonstrated that α-SMA could be detected in the control cells cultured in normal glucose (Figure 7). High glucose elevated the expression of α-SMA by approximately 55%. Similar to the observation *in vivo*, a dose-dependent inhibitory effect of glucosamine on the levels of α-SMA was detected in cultured endothelial cells *in vitro*, under both basal and high glucose conditions, suggesting the possible suppressive role of glucosamine in α-SMA expression in ECs.

## Discussion

As the underlying hypothesis of this study, we demonstrate that glucosamine modulates the initiation and progression of experimental diabetic nephropathy. We found, unexpectedly, that glucosamine reduced mesangial expansion and expression of fibronectin and α-SMA in diabetic nephropathy, and decreased the number of α-SMA+ endothelial cells in the glomeruli, while renal function and expression of inflammatory factors and other genes associated with accumulation of extracellular matrix were not affected by glucosamine.

Mesangial expansion plays an important role in the development of diabetic nephropathy. In the present study we found that the mesangial expansion was more pronounced in diabetic mice. The data are in line with the observation by other studies (4, 27, 28). Increased protein expression of α-SMA, collagen 4 and fibrinectin-1, and CTGF in the diabetic nephropathy was detected concomitantly, supporting previous published studies (27–30). At the mRNA level, we found an increased expression of fibronectin in STZ-induced diabetic kidneys, but no other significant changes in collagens and α-SMA expression in diabetic mice which contradicts some previously reported data (7, 28, 30, 31). Discrepancies among studies might be a result of different study designs, different protocols for diabetes induction, and different durations of hyperglycemia. No obvious morphological changes were detected in diabetic glomeruli compared with controls. Giving the fact that STZ-induced diabetic nephropathy in C57bl6 exhibit mild to moderate renal pathology (32), alteration in PAS may indicate the very early changes in diabetic nephropathy. PAS, which is staining structures containing high amount of carbohydrate macromolecules, highlights basement membranes of glomerular capillaries, mesangial matrix and potential expansion. Thus, increased PAS staining in diabetic glomeruli may indicate a significant potential modification of the composition of the mesangial matrix, and changes of the GBM. In this respect, increased PAS staining in glomeruli is a characteristic feature of the early stages of diabetic nephropathy rather than a typical fibrotic marker in the late stages of DN. Further analysis of extracellular matrix accumulation, such as of fibronectin, supported the data. In this study, we found that glucosamine therapy reduced the mesangial expansion and accumulation of extracellular matrix in diabetic mice. The effect of glucosamine on mesangial expansion in the diabetic nephropathy has not been reported. Glucosamine was proposed to be deteriorative in diabetic nephropathy based on the hypothesis that endogenous glucosamine promotes the activation of the HBP, which is linked to the development of DN. Nevertheless, glucosamine was found to possess an antifibrotic effect in the mouse model of unilateral ureteral obstruction with progressive renal fibrosis. Park et al., demonstrated that the fibrosis displayed by Masson trichrome staining was reduced with a concomitant inhibition of α-SMA expression upon glucosamine treatment (18). Furthermore, an inhibition of other gene expression related to fibrosis except α-SMA, such as fibronectin and collagen, was also shown in that paper. However, in our study, we detected a reduction of fibronectin protein expression in glomeruli, but no effect of glucosamine on the mRNA expression of FN-1, α-SMA, Col 1A1, 3A1, 4A1, 4A3, and CTGF could be determined. An interesting finding in present study is the inhibitory effect of glucosamine on α-SMA expression in control kidneys as well. Differential regulation of fibronectin and collagen 4 by glucosamine may be a result of different molecular characters and the early stage of fibrosis in the glomeruli in the study. Additionally, glucosamine might inhibit the initiation of accumulation of extracellular matrix in diabetic nephropathy as a consequence of maintaining expression of α-SMA at a lower level.

In the current study, glucosamine showed an inhibitory impact on mesangial expansion and expression of fibronectin and α-SMA. Nevertheless, glucosamine is likely not eliminating all ECM components, such as collagen 1, 3 and 4. Several studies showed that expression of extracellular matrix proteins such as α-SMA is controlled by the expression or activity of TGF-β, a key growth factor involved in renal fibrosis. Nevertheless, the data reported on the impact of glucosamine on TGF-β expression or activity are controversial. Park et al., reported an inhibitory effect of glucosamine on the expression of TGF-β expression, while, on the contrary, Kolm-Litty et al., showed that glucosamine increased RNA expression and activity of TGF-β in mesangial cells (5, 18). It seems that cell types, culture conditions, and glucosamine dosage can explain the variation in experimental results. Additionally, in our study, increased expression of CTGF, fibronectin, and collagen 4 was detected in the diabetic kidneys. However, glucosamine affected the expression of fibronectin, but not other extracellular matrix proteins in either non-diabetic or diabetic conditions. It denotes that there might be other possible mechanisms through which glucosamine hampers the accumulation of extracellular matrix in the kidney.

Another interesting finding in the study is that glucosamine lowered not only the expression of α-SMA in diabetic kidneys, but also the number of α-SMA+ endothelial cells. α-SMA is expressed by renal tubular cells, mesangial cells, and endothelial cells. α-SMA has been identified as a marker of myofibroblasts, implicated in Endothelial- and Epithelial-MT in DN. Podocytes in glomeruli may undergo EMT in DN as well. However, we did not detect obvious α-SMA+ podocytes in our study. The role of podocytes in glucosamine-induced improvement of extracellular matrix accumulation needs further investigations. EndoMT has been also described in diabetic nephropathy, including in STZ induced rodent experimental nephropathy in which α-SMA presents as an important marker of their mesenchymal transitions (33). Similar to the report by Zeisberg et al., we observed colocalization of α-SMA and endothelial cell marker CD31, and increased α-SMA expression in diabetic kidneys with elevated amount of α-SMA+ endothelial cells in the diabetic glomeruli. Glucosamine reduced α-SMA expression in eyes in pathological conditions (34, 35). Similar results showing inhibition of α-SMA expression by glucosamine were found in our study in diabetic nephropathy. Interestingly, our *in vitro* experimental data supported the report published by Lu et al. and Spillmann et al. showing that α-SMA is expressed in endothelial cells cultured in basal endothelial culture medium, indicating EndoMT might be a general feature in physiological conditions (36, 37). Furthermore, high glucose promoted the expression of α-SMA in endothelial cells. The results from cultured endothelial cells revealed that glucosamine inhibited endothelial expression of α-SMA in both normal and high glucose conditions, suggesting that EndoMT might be a mechanism contributing to the antifibrotic effect of glucosamine in the diabetic nephropathy. Together with the finding *in vivo*, this suggests that EndoMT might participate in reducing the mesangial expansion in the study. Apart from its role in EndoMT, α-SMA has been also predominantly detected in the mesangial regions in kidney disease (38). Due to the multitudinous effects of glucosamine caused by its characteristics, multiple mechanisms are likely involved in glucosamine-mediated reduction of extracellular matrix accumulation. Whether the results in our study indicate a possible anti-fibrotic activity of glucosamine through other possible mechanisms in the mesangial cells or interstitials needs to be further investigated.

Albuminuria and glomerular hyperfiltration are characteristics of diabetic nephropathy. In the present study, albuminuria and ACR were significantly greater in diabetic mice than in control mice. Our results are in accordance with reports showing an increase in albumin excretion in STZ-induced DN models (32, 39). An influence of exogenous glucosamine on kidney function in diabetic nephropathy has not been described. We demonstrated that therapy with glucosamine did not change the albumin excretion as well as the creatinine clearance, suggesting that glucosamine has no influence on kidney function. An unexpected finding in our study was the reduced mesangial expansion by glucosamine without improvement of urinary albumin excretion. We speculate that the application of glucosamine likely leads to an early change in mesangial expansion. Our results support the reports by Suzuki et al. showing that in the early stage of diabetic nephropathy, the urinary albumin to creatinine ratio induced by diabetes might remained unchanged, for instance upon glucosamine treatment in our study (40). Moreover, fibrosis in diabetic nephropathy can occur prior to, or in spite of, normal albuminuria (41). Our data showing improved mesangial expansion without impact on urinary ACR indicate clearly that there are multiple mechanisms involved in glucosamine effect in DN.

In contrast to published data, we did not find a significant increase in IL-1β and TNF-α expression neither transcriptionally nor translationally in STZ-induced diabetic kidneys in the present study (42, 43). The results of our study indicate that an effect of glucosamine on inflammation in DN would probably be rather weak.

Our data contradict the hypothesis of a causal link between oral glucosamine supplementation, activation of the HBP and the damage of diabetic kidney in the field of diabetes research, suggesting that the oral glucosamine supplementation may hamper hyperglycemia-induced glomerular damage, likely through inhibition of EndoMT instead of an anti-inflammatory effect. However, further studies on its underlying mechanisms are still required.

## Data availability statement

The original contributions presented in this study are included in the article/supplementary material, further inquiries can be directed to the corresponding author.

## Ethics statement

The studies involving human participants were reviewed and approved by Ethics committee (Medical Faculty Mannheim, University of Heidelberg), 2012-338N-MA. The patients/participants provided their written informed consent to participate in this study. The animal study was reviewed and approved by Regierungspräsidium Karlsruhe, G178/15.

## Author contributions

YF designed the study. LT, RE, UT, JW, FS, ML, AA, and YW performed the experiments. LT, RE, H-PH, and YF interpreted the data. LT, RE, and YF wrote the manuscript with input from all authors. H-PH and YF provided essential expertise and knowledge of metabolic diseases for the study. All authors have approved the manuscript for publication.
